# Clinical Outcomes of Metronidazole Monotherapy Versus Metronidazole Plus *Clostridium butyricum* MIYAIRI 588 Combination Therapy in Non-Severe *Clostridioides difficile* Infection: A Retrospective Cohort Study

**DOI:** 10.3390/biotech15020044

**Published:** 2026-06-11

**Authors:** Yota Yamada, Mana Nemoto, Motoyasu Miyazaki, Hitomi Hirata, Konatsu Hosogoe, Akio Nakashima, Hisako Kushima, Hiroshi Ishii, Osamu Imakyure

**Affiliations:** 1 Department of Pharmacy, Fukuoka University Chikushi Hospital, Fukuoka 818-8502, Japan; m.nemoto.ad@adm.fukuoka-u.ac.jp (M.N.); motoyasu@fukuoka-u.ac.jp (M.M.); h.hirata.cd@adm.fukuoka-u.ac.jp (H.H.); anakashima@fukuoka-u.ac.jp (A.N.); imakyure@fukuoka-u.ac.jp (O.I.); 2 Department of Infection Control and Prevention, Fukuoka University Chikushi Hospital, Fukuoka 818-8502, Japan; k.hosogoe.pl@adm.fukuoka-u.ac.jp (K.H.); hkushi@fukuoka-u.ac.jp (H.K.); hishii@fukuoka-u.ac.jp (H.I.); 3 Department of Hospital Pharmacy, Faculty of Pharmaceutical Sciences, Fukuoka University, Fukuoka 814-0180, Japan; 4 Department of Clinical Laboratory, Fukuoka University Chikushi Hospital, Fukuoka 818-8502, Japan; 5 Department of Respiratory Medicine, Fukuoka University Chikushi Hospital, Fukuoka 818-8502, Japan

**Keywords:** *Clostridioides difficile* infection, metronidazole, *Clostridium butyricum* MIYAIRI 588, adjunctive therapy

## Abstract

*Clostridioides difficile* infection (CDI) remains a major healthcare-associated infection, and metronidazole (MNZ) is still used for selected patients with non-severe infection in Japan. Although *Clostridium butyricum* MIYAIRI 588 (CBM588) may be used as an adjunctive therapy, clinical evidence regarding its add-on value to MNZ in non-severe CDI remains limited. We conducted an exploratory single-center retrospective cohort study of adults with non-severe CDI treated with MNZ between April 2015 and March 2025. Of the 161 patients diagnosed with *C. difficile* infection, 53 patients met the eligibility criteria and were analyzed. Patients were categorized into MNZ monotherapy (*n* = 27) or MNZ plus CBM588 combination therapy groups (*n* = 26), and clinical outcomes were compared. Clinical cure was achieved in 51.9% of patients in the monotherapy group and 69.2% of patients in the combination therapy group; however, the difference was not statistically significant (*p* = 0.196). Recurrence was uncommon in both groups (3.7% vs. 0%), and exploratory multivariable analysis revealed that CBM588 use was not independently associated with clinical cure. These findings do not establish a definitive benefit of adjunctive CBM588 therapy and should be interpreted cautiously as hypothesis-generating given the limited sample size and non-significant result.

## 1. Introduction

*Clostridioides difficile* infection (CDI) is a major healthcare-associated infection and has substantial effects on patient outcomes and healthcare resources owing to its frequent recurrence and resulting prolonged hospital stays and increased healthcare costs [1,2,3]. Current international guidelines emphasize that key components of CDI management include selecting appropriate therapeutic agents according to disease severity and implementing strategies that prevent recurrence [4,5,6]. Although recent international guidelines increasingly recommend fidaxomicin and vancomycin over metronidazole (MNZ), Japanese guidance continues to include MNZ as a treatment option for selected patients with non-severe CDI [4,5,6,7]. Therefore, the present study was not designed to compare MNZ with other recommended first-line agents, such as fidaxomicin or vancomycin, or to support MNZ-based therapy over these alternatives. Rather, we focused on MNZ because it remains used in routine clinical practice in Japan for selected patients with non-severe CDI [7,8].

Altered intestinal microbiota is a key feature in CDI pathogenesis, and thus interest in adjunctive therapeutic strategies aimed at restoring gut microbial homeostasis is growing [1,2,3]. In addition to direct antimicrobial treatment against *C. difficile*, interventions that help re-establish colonization resistance may be clinically relevant. Indeed, loss of microbial diversity and impaired short-chain fatty acid production are thought to contribute to the persistence and progression of CDI [1,2,3]. Among such strategies, *Clostridium butyricum* MIYAIRI 588 (CBM588), a butyrate-producing probiotic widely used in Japan, has attracted particular interest. Experimental studies have suggested that CBM588 may enhance colonization resistance against *C. difficile*, modulate the intestinal environment in a favorable manner, and attenuate *C. difficile*-associated cytotoxicity [9,10]. Despite these findings, clinical evidence supporting the use of CBM588 in CDI remains limited.

A previous retrospective study in adults with mild-to-moderate CDI suggested that *C. butyricum*, either alone or in combination with MNZ, achieved treatment success rates comparable to those of MNZ monotherapy [11]. However, that study evaluated *C. butyricum* therapy in a broader treatment context and was not designed to isolate the additional contribution of CBM588 when added to MNZ [11]. Other probiotics have also been evaluated as adjunctive therapies to standard anti-CDI antibiotics, including MNZ or vancomycin; however, these studies used different probiotic formulations, concomitant anti-CDI therapies, and patient populations [12,13,14]. Thus, clinical evidence specifically addressing the add-on value of CBM588 to MNZ in patients with non-severe CDI remains limited. This issue is particularly relevant in Japan, where severity-based treatment selection is reflected in domestic guidance and clinical outcomes of CDI have been evaluated according to disease severity [7,8]. Because MNZ remains an accepted option for selected patients with non-severe CDI in Japan, this population provides a clinically relevant setting in which to explore the potential add-on value of CBM588 under MNZ therapy.

Therefore, in this exploratory single-center retrospective cohort study conducted in Japan, we compared clinical outcomes, including clinical cure and recurrence, between MNZ monotherapy and MNZ plus CBM588 combination therapy in patients with non-severe CDI. We also explored patient- and treatment-related factors associated with clinical cure in this population. This study was intended to generate clinical hypotheses regarding adjunctive CBM588 use during MNZ therapy and was not designed to establish its definitive benefit or to compare MNZ-based therapy with fidaxomicin- or vancomycin-based treatment.

## 2. Materials and Methods

### 2.1. Study Design and Patient Care

**Figure 1:**
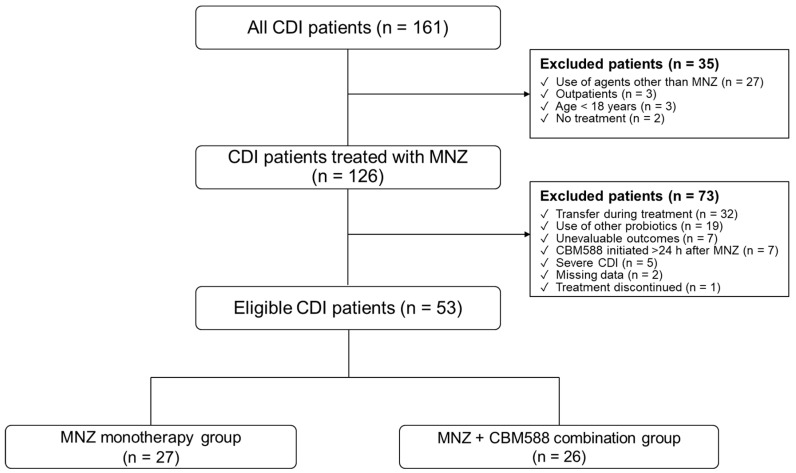
Flow diagram of patient selection.

This exploratory single-center retrospective cohort study was conducted at Fukuoka University Chikushi Hospital between April 2015 and March 2025. Hospitalized patients aged 18 years or older who were diagnosed with CDI and treated with MNZ during the study period were screened for eligibility. Of the 161 patients diagnosed with CDI, 53 patients were included in the final analysis (Figure 1). Because this was an exploratory retrospective study including all eligible patients during the predefined study period, an a priori sample size calculation was not performed.

The exclusion criteria included an age of less than 18 years, transfer to another hospital during treatment, unevaluable clinical outcomes, use of probiotics other than CBM588, initiation of CBM588 more than 24 h after MNZ initiation, missing data, treatment discontinuation, and severe CDI. The combination therapy group was defined as patients who received CBM588 at the time of MNZ initiation or within 24 h thereafter. The decision to prescribe adjunctive CBM588 was made at the discretion of the attending physician. No standardized institutional protocol specified the dose or duration of adjunctive CBM588 therapy during the study period, and these were determined by the attending physician according to routine clinical practice. Patients were classified into an MNZ monotherapy group (*n* = 27) and an MNZ plus CBM588 combination therapy group (*n* = 26). For patients with multiple CDI episodes, only the first episode was included in the analysis. This study was approved by the Ethics Committee of Fukuoka University School of Medicine (C25-04-006).

### 2.2. CDI Diagnosis and Severity Assessment

The diagnosis of CDI was based on clinical symptoms and stool test results according to institutional practice during the study period. The diagnostic approach to CDI differed before and after the introduction of the nucleic acid amplification test (NAAT; Xpert^®^ *C. difficile*; Cepheid, Sunnyvale, CA, USA) at our institution. Before and after the introduction of NAAT, glutamate dehydrogenase (GDH) and toxin levels were assessed using the rapid membrane enzyme immunoassay C. DIFF QUIK CHEK COMPLETE^®^ (TechLab, Blacksburg, VA, USA). NAAT was introduced at our institution in April 2020, and thereafter, GDH-positive/toxin-negative cases were additionally evaluated by NAAT. When the diagnosis of CDI could not be determined solely from stool test results, a comprehensive assessment of clinical findings, such as diarrhea, fever, and abdominal pain, was performed. Even if patients were positive for both GDH and toxin, they were not considered to have CDI if the attending physician judged colonization or another disease to be more likely.

Disease severity was assessed using the MN criteria [15,16]. In this scoring system, age, abdominal symptoms, body temperature, diarrhea frequency, white blood cell count, estimated glomerular filtration rate (eGFR), serum albumin levels, and imaging findings are each scored with 0–3 points, and the total score is used to classify disease severity as mild (0–4), moderate (5–9), severe (10–13), or critical (14–19). In the present study, mild and moderate cases were categorized as non-severe CDI, whereas severe and critical cases were categorized as severe CDI. Only patients with non-severe CDI were included in the present analysis.

### 2.3. Clinical Characteristics

Clinical and demographic data were collected retrospectively from electronic medical records. The evaluated variables were age, sex, H2 receptor blocker use, proton pump inhibitor (PPI) use, immunosuppressant use, prior antibiotic exposure, presence of inflammatory bowel disease (IBD), Charlson Comorbidity Index (CCI) score [17], white blood cell count (WBC), eGFR, and serum albumin level.

### 2.4. Clinical Outcomes

The primary outcome was clinical cure. Secondary outcomes included recurrence, treatment duration, and hospital stay length. Clinical cure was defined as the completion of CDI-directed therapy without treatment escalation and with resolved diarrhea for at least two consecutive days by the end of treatment. Recurrence was defined as the reappearance of CDI within 8 weeks after the previous episode.

### 2.5. Statistical Analysis

Categorical and continuous variables are presented as number (%) and median (interquartile range), respectively. For univariate analyses, the chi-square test or Fisher’s exact test were used for categorical variables, and the Mann–Whitney U test was used for continuous variables, as appropriate. To assess factors independently associated with clinical cure, variables with *p* < 0.20 in the univariate analysis were entered into a multivariate logistic regression model to avoid excluding potentially relevant confounders in the small dataset. Based on this criterion, WBC, PPI use, and CBM588 use were included in the multivariate analysis. Because of the limited sample size and the small number of outcome events, additional adjustment methods such as propensity score-based analyses were not performed. Therefore, baseline imbalances were described and considered as limitations when interpreting the findings. Odds ratios and 95% confidence intervals were calculated. For WBC, the odds ratio was expressed per 1000/µL increase. A two-sided *p* value of <0.05 was considered statistically significant. All statistical analyses were performed using JMP^®^ version 10 (SAS Institute Inc., Cary, NC, USA).

## 3. Results

### 3.1. Patient Characteristics

A total of 53 patients were included in the final analysis, which included a MNZ monotherapy (*n* = 27) and MNZ plus CBM588 combination therapy group (*n* = 26). In the overall cohort, the median age was 79.0 years (66.0–84.5), and 29 patients (54.7%) were male. The overall cohort included patients who were diagnosed after the introduction of NAAT (*n* = 21; 39.6%), received H2 receptor blockers (*n* = 2; 3.8%), received immunosuppressants (*n* = 7; 13.2%), received PPIs (*n* = 30; 56.6%), underwent prior antibiotic exposure (*n* = 49; 92.5%), and had IBD (*n* = 11; 20.8%). The median CCI score was 1.0 (0.0–2.0), the median WBC was 9200/µL (7000–12,600), the median estimated eGFR was 75.4 mL/min/1.73 m^2^ (45.4–116.4), and the median albumin level was 2.3 g/dL (2.0–2.7). Although most baseline characteristics did not differ significantly between the two treatment groups, several significant baseline differences were identified (Table 1). Patients in the MNZ plus CBM588 group were more frequently diagnosed after the introduction of NAAT and had higher serum albumin levels, whereas those in the MNZ monotherapy group had higher estimated eGFR.

### 3.2. Treatment Details and Clinical Outcomes

The median daily dose of MNZ was 1500 mg/day (1000–1500) in the MNZ monotherapy group and 1250 mg/day (1000–1500) in the MNZ plus CBM588 group (*p* = 1.000). MNZ treatment duration did not differ significantly between the MNZ monotherapy and MNZ plus CBM588 groups (Table 2). In the combination therapy group, the median daily dose of CBM588 was 120 mg/day (60–120). Clinical cure was observed in 14 patients (51.9%) in the MNZ monotherapy group and in 18 patients (69.2%) in the MNZ plus CBM588 group; however, the difference was not statistically significant (Table 2). Recurrence was observed in one patient in the MNZ monotherapy group and in no patients in the MNZ plus CBM588 group (Table 2). No adverse events clearly attributable to CBM588 were documented in the available medical records.

### 3.3. Factors Associated with Clinical Cure

PPI use, CBM588 use, and WBC met the prespecified criterion (*p* < 0.20) for inclusion in the multivariate analysis. These variables were entered into a multivariate logistic regression model, which showed that CBM588 and WBC were not independently associated with clinical cure. PPI use was not significantly associated with clinical cure in the multivariate analysis (Table 3).

## 4. Discussion

In this exploratory retrospective cohort study of patients with non-severe CDI, the clinical cure rate differed between the groups; however, the difference was not statistically significant. Exploratory multivariable analysis did not show an independent association between CBM588 use and clinical cure. These findings do not establish a definitive benefit of adjunctive CBM588 therapy and should be interpreted cautiously as hypothesis-generating.

A possible role for CBM588 as an adjunctive therapy is biologically plausible in CDI. CDI develops in the setting of altered gut microbiota, and loss of colonization resistance after antibiotic exposure is thought to contribute to disease onset and recurrence [1,2,3]. Potential mechanisms underlying the adjunctive role of CBM588 may include restoration of colonization resistance, modulation of gut microbial metabolism, and attenuation of C. difficile-associated cytotoxicity [9,10]. In experimental CDI models, CBM588 has been reported to enhance colonization resistance through metabolic and immune modulation, including changes in gut succinate levels and host immune responses [9]. In addition, in vitro data have shown that CBM588 can reduce or eliminate the cytotoxic effect of *C. difficile* in co-culture experiments [10]. As a butyrate-producing probiotic, CBM588 may also contribute to intestinal homeostasis through short-chain fatty acid production and support of epithelial barrier function [18]. However, these mechanisms remain largely experimental and were not directly evaluated in the present retrospective study. Lee et al. reported that *C. butyricum*, either alone or in combination with MNZ, achieved treatment success comparable to that of MNZ monotherapy in adults with mild-to-moderate CDI [11]. The present study adds to this literature by focusing specifically on non-severe CDI treated with MNZ and by examining CBM588 use in routine clinical practice in Japan. In our cohort, CBM588 use was more common among patients who achieved clinical cure. However, exploratory multivariable analysis did not demonstrate an independent association between CBM588 use and clinical cure, and the wide confidence interval precluded any meaningful inference regarding effect size. The present study also provides clinical data from a real-world Japanese setting, in which MNZ is still used for selected patients with non-severe CDI. The susceptibility of CBM588 to metronidazole is an important consideration when interpreting the biological plausibility of adjunctive CBM588 therapy. Previous in vitro data reported metronidazole MIC values of 1.0–2.0 mg/L for CBM588, indicating that CBM588 should not be regarded as metronidazole-resistant or completely unaffected by metronidazole in a strict susceptibility-testing sense [19]. A recent Japanese report also showed metronidazole MIC values of ≤ 2 mg/L for three medicinal CBM588 strains from different lots [20]. These findings support the need for caution when assuming that CBM588 remains viable during MNZ therapy. However, CBM588 is a spore-forming probiotic bacterium, and in vitro susceptibility data based on vegetative bacterial growth may not fully reflect its potential viability under intestinal conditions [19]. Experimental mouse data have shown that *C. butyricum* can be detected in feces under concomitant administration with anti-CDI antibiotics, including metronidazole [21]. Nevertheless, these findings do not establish persistent colonization of CBM588 during metronidazole therapy in human CDI. Therefore, whether CBM588 remains viable or colonizes the intestine during MNZ therapy requires confirmation in future prospective microbiological studies.

Any interpretation of these findings must consider the changes in diagnostic practice during the study period, during which NAAT was introduced. Patients diagnosed after its introduction were more frequently included in the combination therapy group. Although the analysis was restricted to patients with non-severe CDI, the introduction of NAAT may have altered case ascertainment. This temporal imbalance raises the possibility that differences in diagnostic sensitivity and case definition, rather than treatment effects alone, may have contributed to the observed between-group differences. In addition to this temporal imbalance, some baseline characteristics differed between the treatment groups. The MNZ plus CBM588 group had higher serum albumin levels, whereas the MNZ monotherapy group had higher estimated eGFR. These differences may reflect underlying variations in patient condition, nutritional status, renal function, or physician treatment selection. Because adjunctive CBM588 use was not randomized, the observed difference in clinical cure should be interpreted cautiously in light of potential residual confounding.

This issue remains clinically relevant because MNZ is still used in selected patients with non-severe CDI in routine practice, even though recent international guidelines increasingly favor fidaxomicin or vancomycin over MNZ [4,5,6,7]. The present study reflects a transitional period in CDI management in Japan, in which MNZ remains an accepted option for selected patients with non-severe CDI despite an international shift toward fidaxomicin- or vancomycin-based strategies. In this context, adjunctive strategies aimed at restoring colonization resistance remain of interest. Previous studies have evaluated probiotics as adjunctive therapies to standard anti-CDI antibiotics, including metronidazole or vancomycin. However, these studies used different probiotic formulations, concomitant anti-CDI therapies, and patient populations, and none specifically evaluated the add-on value of CBM588 to MNZ in patients with non-severe CDI [12,13,14]. Interventions aimed at restoring gut microbiota have attracted particular attention in recurrent CDI [22]. Microbiota restoration therapies, such as fecal microbiota transplantation and microbiota-based live biotherapeutic products, are primarily used after standard-of-care antibiotics to prevent recurrent CDI [22,23,24,25]. Conversely, CBM588 represents a simpler adjunctive approach that can be used concurrently with anti-CDI treatment; however, its role in improving initial treatment response remains uncertain. Adjunctive therapies such as CBM588 may also be relevant to recurrence prevention through their effects on gut microbiota. However, in this study, recurrence occurred in only one patient, and thus recurrence-related outcomes could not be adequately assessed. The low recurrence rate may in part reflect the restriction of the analysis to patients with non-severe CDI, as severe CDI is associated with recurrence [26,27].

PPI use was more common among patients who did not achieve a clinical cure; however, this association was not statistically significant in the exploratory analysis. Previous studies have mainly linked PPI use to recurrent CDI, and some have also reported an association with poorer treatment responses [28,29,30,31]. Gastric acid suppression may perturb gastrointestinal microbiota and diminish the protective effect of gastric acid, and thus PPI exposure may affect treatment responses in CDI [32,33]. Although causality cannot be inferred, this finding may reflect a broader disruption of gastrointestinal homeostasis associated with acid suppression. In the present study, only patients with non-severe CDI were included, which most likely decreased the heterogeneity in disease severity. Nevertheless, patients receiving PPIs may still have differed in background characteristics other than severity, and the current findings do not establish that PPI use itself was responsible for the observed association.

This study has several limitations. First, this was an exploratory single-center retrospective study with a small sample size, resulting in limited statistical power to detect between-group differences. Because of the limited sample size, the multivariable analysis should be interpreted cautiously as exploratory. Therefore, the present findings should not be interpreted as evidence establishing a definitive benefit of adjunctive CBM588 therapy. Second, treatment selection was not randomized, and the decision to prescribe adjunctive CBM588 was left to the attending physician. As a result, unmeasured differences in patient background characteristics or clinical judgment may have influenced the choice for combination therapy, raising the possibility of confounding by indication. Third, temporal imbalance related to the introduction of NAAT and baseline differences, including estimated eGFR and serum albumin levels, may have affected case ascertainment and treatment response, thereby contributing to residual confounding. Fourth, recurrence occurred in only one patient, precluding meaningful evaluation of the effect of CBM588 on recurrence. Fifth, no microbiological, strain-level, or mechanistic analyses were performed. In particular, we did not directly evaluate the persistence, viability, or intestinal colonization of CBM588 during MNZ therapy. We did not isolate CBM588 from stool samples during treatment or perform molecular confirmation of strain persistence. Therefore, the present study cannot determine whether CBM588 remained viable during MNZ therapy or whether it contributed mechanistically to the observed clinical outcomes. Future prospective studies should incorporate microbiological or molecular assessments, such as stool culture and strain-specific identification, to clarify the persistence or colonization of CBM588 during MNZ therapy. In addition, biomarker-based approaches may help better characterize patient heterogeneity, improve patient stratification, and support more individualized treatment strategies for CDI. Sixth, although no adverse events clearly attributable to CBM588 were documented in the available medical records, adverse events were not systematically monitored or predefined as study outcomes. Therefore, adverse events and tolerability could not be reliably compared between the two treatment groups. Finally, this study was conducted at a single Japanese center and included only patients with non-severe CDI treated with MNZ; therefore, our findings may not be generalizable to other healthcare settings, patients with severe CDI, or those treated with other first-line agents. Collectively, these retrospective design-related limitations may have affected the internal validity of the findings and preclude causal inference.

In conclusion, in this exploratory single-center retrospective cohort study of patients with non-severe CDI, no statistically significant difference in clinical cure was observed between the MNZ monotherapy and MNZ plus CBM588 groups. Given the limited sample size, potential confounding, and lack of direct microbiological confirmation of CBM588 persistence during MNZ therapy, these findings do not establish a definitive benefit of adjunctive CBM588 therapy and should be interpreted cautiously as hypothesis-generating. Larger prospective studies incorporating microbiological or molecular assessments of CBM588 persistence, together with biomarker-based approaches for patient stratification, are needed to clarify whether adjunctive CBM588 has a role during MNZ therapy in selected patients with non-severe CDI in Japan.

## Figures and Tables

**Table 1:** Baseline characteristics of patients with non-severe CDI treated with MNZ monotherapy or MNZ plus CBM588.

Variable	MNZ Monotherapy (*n* = 27)	MNZ + CBM588 (*n* = 26)	*p* Value
Age, years *	81.0 (71.0–86.0)	75.5 (43.8–84.0)	0.162
Male sex, *n* (%)	13 (48.2)	16 (61.5)	0.327
After introduction of NAAT, *n* (%)	7 (25.9)	14 (53.8)	0.038
Charlson Comorbidity Index score *	1.0 (0.0–2.0)	2.0 (1.0–3.0)	0.188
Inflammatory bowel disease, *n* (%)	3 (11.1)	8 (30.8)	0.099
H2 receptor blocker use, *n* (%)	1 (3.7)	1 (3.9)	1.000
Proton pump inhibitor use, *n* (%)	16 (59.3)	14 (53.9)	0.691
Immunosuppressant use, *n* (%)	3 (11.1)	4 (15.4)	0.704
Prior antibiotic exposure, *n* (%)	26 (96.3)	23 (88.5)	0.351
White blood cell count, /µL *	9000 (6600–12,500)	9600 (7825–12,900)	0.682
Estimated glomerular filtration rate, mL/min/1.73 m^2^ *	92.8 (48.0–121.8)	64.2 (37.4–87.3)	0.029
Albumin, g/dL *	2.2 (2.0–2.6)	2.4 (2.2–3.0)	0.024

Categorical variables are presented as *n* (%), and continuous variables are presented as median (interquartile range) and marked with an asterisk. Abbreviations: CBM588, *Clostridium butyricum* MIYAIRI 588; CDI, *Clostridioides difficile* infection; MNZ, metronidazole; NAAT, nucleic acid amplification test.

**Table 2:** Clinical outcomes of patients with non-severe CDI treated with MNZ monotherapy or MNZ plus CBM588.

Variable	MNZ Monotherapy (*n* = 27)	MNZ + CBM588 (*n* = 26)	*p* Value
Treatment duration, days *	11.0 (7.0–14.0)	11.0 (7.7–14.0)	0.993
Length of hospital stay, days *	42.0 (25.0–53.0)	31.5 (26.0–47.3)	0.545
Clinical cure, *n* (%)	14 (51.9)	18 (69.2)	0.196
Recurrence, *n* (%)	1 (3.7)	0 (0.0)	1.000

Categorical variables are presented as *n* (%), and continuous variables are presented as median (interquartile range) and marked with an asterisk. Abbreviations: CBM588, *Clostridium butyricum* MIYAIRI 588; CDI, *Clostridioides difficile* infection; MNZ, metronidazole.

**Table 3:** Factors associated with clinical cure in patients with non-severe CDI.

Variable	No Clinical Cure (*n* = 21)	Clinical Cure (*n* = 32)	Univariate *p* Value	Adjusted OR	95% CI	Multivariate *p* Value
Age, years *	80.0 (72.5–86.0)	74.5 (55.8–84.0)	0.270	—	—	—
Male sex, *n* (%)	11 (52.4)	18 (56.3)	0.782	—	—	—
After introduction of NAAT, *n* (%)	9 (42.9)	12 (37.5)	0.697	—	—	—
Charlson Comorbidity Index score *	1.0 (0.0–2.0)	1.0 (1.0–3.0)	0.416	—	—	—
Inflammatory bowel disease, *n* (%)	3 (14.3)	8 (25.0)	0.493	—	—	—
H2 receptor blocker use, *n* (%)	0 (0)	2 (6.3)	1.000	—	—	—
Proton pump inhibitor use, *n* (%)	15 (71.4)	15 (46.9)	0.077	0.36	0.10–1.15	0.086
Immunosuppressant use, *n* (%)	1 (4.8)	6 (18.8)	0.228	—	—	—
CBM588 use, *n* (%)	8 (38.1)	18 (56.3)	0.196	2.05	0.65–6.79	0.218
Prior antibiotic exposure, *n* (%)	20 (95.2)	29 (90.6)	1.000	—	—	—
White blood cell count, /µL *	8700 (6300–10,450)	9950 (8100–13,350)	0.148	1.03	0.91–1.18	0.601
Estimated glomerular filtration rate, mL/min/1.73 m^2^ *	74.6 (40.4–131.9)	75.5 (51.6–111.6)	0.978	—	—	—
Albumin, g/dL *	2.4 (2.05–2.95)	2.2 (2.0–2.6)	0.553	—	—	—

Categorical variables are presented as *n* (%), and continuous variables are presented as median (interquartile range) and marked with an asterisk. Abbreviations: CBM588, *Clostridium butyricum* MIYAIRI 588; CDI, *Clostridioides difficile* infection; CI, confidence interval; MNZ, metronidazole; NAAT, nucleic acid amplification test; OR, odds ratio.

## Data Availability

The original contributions presented in this study are included in the article. Further inquiries can be directed to the corresponding author.

## Abbreviations

The following abbreviations are used in this manuscript:

CDI	*Clostridioides difficile* infection
MNZ	metronidazole
CBM588	*Clostridium butyricum* MIYAIRI 588
NAAT	nucleic acid amplification test
GDH	glutamate dehydrogenase
PPI	proton pump inhibitor
IBD	inflammatory bowel disease
CCI	Charlson Comorbidity Index
WBC	white blood cell count
eGFR	estimated glomerular filtration rate
OR	odds ratio
CI	confidence interval
